# Development of a new category system for the profile morphology of temporomandibular disorders patients based on cephalograms using cluster analysis

**DOI:** 10.3389/fpubh.2022.1045815

**Published:** 2022-11-17

**Authors:** Rui Zhu, Yun-Hao Zheng, Zi-Han Zhang, Pei-Di Fan, Jun Wang, Xin Xiong

**Affiliations:** ^1^The State Key Laboratory of Oral Diseases and National Clinical Research Center for Oral Diseases, Department of Prosthodontics, West China Hospital of Stomatology, Sichuan University, Sichuan, China; ^2^The State Key Laboratory of Oral Diseases and National Clinical Research Center for Oral Diseases, Department of Orthodontics, West China Hospital of Stomatology, Sichuan University, Sichuan, China; ^3^Department of Temporomandibular Joint, West China Hospital of Stomatology, Sichuan University, Sichuan, China

**Keywords:** temporomandibular disorders, cluster analysis, cephalometric analysis, classification and regression tree (CART), morphological category

## Abstract

**Objective:**

This study aims to develop a new category scheme for the profile morphology of temporomandibular disorders (TMDs) based on lateral cephalometric morphology.

**Methods:**

Five hundred and one adult patients (91 males and 410 females) with TMD were enrolled in this study. Cluster tendency analysis, principal component analysis and cluster analysis were performed using 36 lateral cephalometric measurements. Classification and regression tree (CART) algorithm was used to construct a binary decision tree based on the clustering results.

**Results:**

Twelve principal components were discovered in the TMD patients and were responsible for 91.2% of the variability. Cluster tendency of cephalometric data from TMD patients were confirmed and three subgroups were revealed by cluster analysis: (a) cluster 1: skeletal class I malocclusion; (b) cluster 2: skeletal class I malocclusion with increased facial height; (c) cluster 3: skeletal class II malocclusion with clockwise rotation of the mandible. Besides, CART model was built and the eight key morphological indicators from the decision tree model were convenient for clinical application, with the prediction accuracy up to 85.4%.

**Conclusion:**

Our study proposed a novel category system for the profile morphology of TMDs with three subgroups according to the cephalometric morphology, which may supplement the morphological understanding of TMD and benefit the management of the categorical treatment of TMD.

## Introduction

Temporomandibular disorders (TMDs) are a set of clinical conditions associated with the temporomandibular joint (TMJ), masticatory muscles, and orofacial structures (1–4). Generally, approximately 5% of the population suffered from these disorders with a prevalence between 5 and 15% in adults (5, 6). However, the situation of TMDs is not encouraging recently. Evidence shows that the prevalence of TMDs is increasing recently, with an overall prevalence of 31% in adults and 11% in children and adolescence (7). Besides, the most frequent TMD related symptoms including restricted mouth opening, TMJ sounds, and TMJ pain have been up to 50% in adults (8), which greatly affects the patients' quality of life.

Nowadays, in spite of various methods with well diagnostic reliability and validity developed for diagnosing TMDs (9–11), the Diagnostic Criteria for Temporomandibular Disorders (DC/TMD) is still the most widely utilized, thorough and accurate diagnostic criteria worldwide for assessment and classification of TMD (12), which comprehensively takes both characterization of the disease in the joint and muscle (Axis I) and psychosocial disability (Axis II) into consideration (13). Although DC/TMD is an excellent tool to diagnose and classify the TMDs, there also exists several vacancies about lateral cephalograms and further efforts are still needed for relevant research.

Lateral cephalometric radiograph, an easily accessible and non-invasive examination, can supply abundant data concerning the cranial, facial bony and soft tissue structures. For its economy and convenience, lateral cephalometric radiograph has been not only widely used as facial analysis before and after orthodontic treatment, but also utilized to explore the association between TMD including its symptoms and the characteristics of craniofacial morphology (14–17). Already in 1995, lateral cephalometry was applied to investigate the association between morphologic features and internal derangements of the temporomandibular joint (15). Recently, the craniofacial morphology of TMD and has been well-investigated (16) and it is reported that patients with TMD exhibit specific craniofacial features compared to patients without TMD (16, 17). Our previous study (14) also validated the results and further observed a significant difference in Frankfort-mandibular plane angle (FMA) between patients with and without TMDs. Besides, we found there existed specific craniofacial features between TMD patients with and without TMJ pain as well (14). At present, although these studies revealed the significant relationship between TMD and morphologic features, the indicators from lateral cephalometric radiograph were still mainly applied to judge the skeletal pattern of the patients by orthodontic diagnosis and only partially reflected the features of TMD, which might help little for the treatment of TMD patients. Consequently, it is necessary to develop a new category system specific to TMD to integrate those significant features for clinical application.

Clustering analysis is an unsupervised learning model widely used in data mining (18) and has been utilized to determine the subtypes of many diseases according to their numerous indicators such as idiopathic inflammatory myopathies (19), class III malocclusion (20) and others (21). However, there was no clustering analysis based on the cephalograms in the research of TMD.

In this study, in order to make the most of these indicators from lateral cephalometric radiograph, we develop a new category system for the profile morphology of TMD patients using cluster analysis according to thirty-six cephalometric parameters.

## Materials and methods

### Subjects and study design

The research was conducted at the Department of Orthodontics, West China Hospital of Stomatology, Sichuan University, from June 2021 to October 2021. All patients were investigated and diagnosed by one TMD specialist who had received extensive training and calibration in the use of the DC/TMD (12).

The inclusion criteria were as follows: (a) patients diagnosed with TMD for the first time; (b) patients aged 18 years or above; and (c) patients with available chart, lateral cephalograms, and photographs. The exclusion criteria were: (a) presence of tumor, trauma and/or surgery history in the maxilla and facial area; (b) presence of clefts and other craniofacial anomalies.

The study was approved by the Ethics Committee of West China School of Stomatology of Sichuan University (Ethics number: 2021-396) and was conducted in accordance with the Declaration of Helsinki. Informed consents were provided with all the patients.

**Figure 1:**
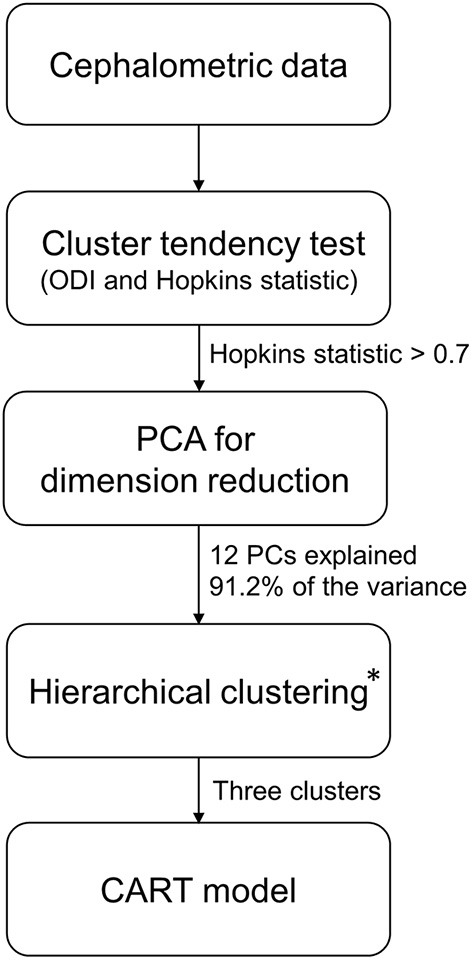
Flowchart for cluster analysis. PCA, principal component analysis; PCs, principal components; CART, classification and regression tree. *Hierarchical clustering and three clusters were optimal clustering algorithm and number of clusters according to the methods in the article.

This study was carried out based on multiple clustering approaches and general procedures were given in the flowchart (Figure 1).

### Cephalometric analysis

All the patients' lateral cephalograms were collected before they started to receive orthodontic treatment by the same radiologist. Patients had to maintain the natural head position with the mandible in the maximum intercuspal position by request (22). The Uceph software (Chengdu Yaxun, Chengdu, China) was applied for cephalometric analysis after collecting the lateral cephalograms.

Table 1 showed the thirty-six cephalometric parameters measured in the study. The measurements were conducted by two researchers blinded to the patients' details. According to the approach described by Xiong et al. (23), inter-observer and intra-observer reliability were examined to ensure the accuracy of the measurements. For inter-observer reliability, 20 lateral cephalograms were selected randomly and measured by the examiners for the first time. After a washout period of 4 weeks, the observer repeated the measurement. The intra-class correlation coefficient (ICC) was calculated to test the repeatability of the results. The examiners were eligible when ICC was over 0.75.

**Table 1:** Cephalometric variables.

**Cranial base**	S-Go (mm)	Interincisal angle (U1-L1) (°)
Saddle/Sella angle (°)	Mandibular body length (Go-Me) (mm)	U1-SN(°)
Anterior cranial base (S-N) (mm)	**Intermaxillary**	UPDH (U6-PP) (mm)
Posterior cranial base (S-Ar) (mm)	Midface length (Co-A) (mm)	LPDH (L6-MP) (mm)
**Maxilla**	ANB (°)	U1-ANS (mm)
SNA (°)	Y-axis (°)	L1-Me (mm)
PP-FH (°)	Y-axis length (mm)	MP-OP (°)
**Mandible**	Wits appraisal (mm)	PP-OP (°)
SNB (°)	Anterior face height (N-Me) (mm)	OP-FH (°)
Gonial/Jaw angle (Ar-Go-Me)(°)	FMA (FH-MP) (°)	Overbite (mm)
Ramus height (Ar-Go) (mm)	ANS-Xi-Pm (°)	Overjet (mm)
Articular angle (S-Ar-Go) (°)	**Dental**	**Soft Tissue**
Dc-Xi-Pm (°)	IMPA (L1-MP) (°)	Upper lip to E-plane (UL-EP) (mm)
SN-MP (°)	FMIA (L1-FH) (°)	Lower lip to E-plane (LL-EP) (mm)

Boldface indicates six categories of the thirty-six cephalometric parameters.

### Cluster tendency analysis

The dissimilarity matrix based on Euclidean distance metrics between the normalized samples was calculated and reordered to form an ordered dissimilarity image (ODI). The visual assessment of cluster tendency algorithm (VAT) was used to visualize the ODI (24). Considering that clustering algorithms will locate and specify clusters in data even if none are present, Hopkins statistic *H* was used to validate cluster tendency. The significance level was set to *H* > 0.7, which meant that data had a cluster tendency and the clustering results were meaningful (25).

### Principal components analysis

Principal components (PCs) are a series of mutually orthogonal variables formed by linear combinations of the original data variables and are arranged in descending order according to their ability to describe the variance of the original data.

To calculate the principal components, the data matrix needed to be normalized first, and the variables of the normalized data matrix *Z* are then linearly combined as principal components in the form of equation (1) through algorithms (e.g., maximum projection variance, singular value decomposition, etc.) making the data have the largest variance in the first principal component, followed by the second principal component, and so on.


(1) $$\begin{array}{l} \begin{array}{c} PC_{k}=\sum_{i=1}^{N}a_{ik}Z_{i} \end{array}\; \end{array}$$

where *PC*_*k*_ is the *k*-th principal component, *a*_*ik*_ is the coefficient of the linear combination obtained according to a specific algorithm, and *Z*_*i*_ is the *i*-th column of the centralized matrix *Z*, i.e., the *i*-th variable.

The first *n* principal components are selected to satisfy (i) the cumulative percentage of variance exceeds 90%; (ii) the (*n*+1)-th to *N*-th principal components have sufficiently small contribution to the variance to be used as pre-processed data for modeling.

### Optimization of number of clusters and clustering algorithm

The number of clusters was evaluated by using 26 indices such as CH index and Dula index, and the optimal number of clusters was selected according to the “ majority voting” principle (26). The optimal clustering algorithm was selected by calculating the connectivity, Dunn and Silhouette indices of three common clustering methods, namely hierarchical clustering, K-means clustering and partitioning around medoids (PAM), for the selected number of clusters.

### Hierarchical clustering on principal components

Hierarchical clustering was performed based on Ward's minimum variance method on the basis of principal component analysis (PCA), and the initial partitions obtained from the hierarchical clustering were improved by K-means clustering (27). The PCA step can be considered as a denoising step which can lead to a more stable clustering.

### Classification and regression tree

Classification and regression tree (CART) algorithm was used to construct a binary decision tree to help dentists classify TMD according to patients' cephalometric characteristics easily. We performed cross-validation to select the optimal tree and performed multiple runs to avoid overfitting. Cephalometric dataset was split into 70% as training set and 30% as validation set and the classification tree model was evaluated by the accuracy of prediction. Confusion matrix was made to visualize and summarize the performance of the CART model (Supplementary Figure 1).

### Statistical analysis

**Figure 2:**
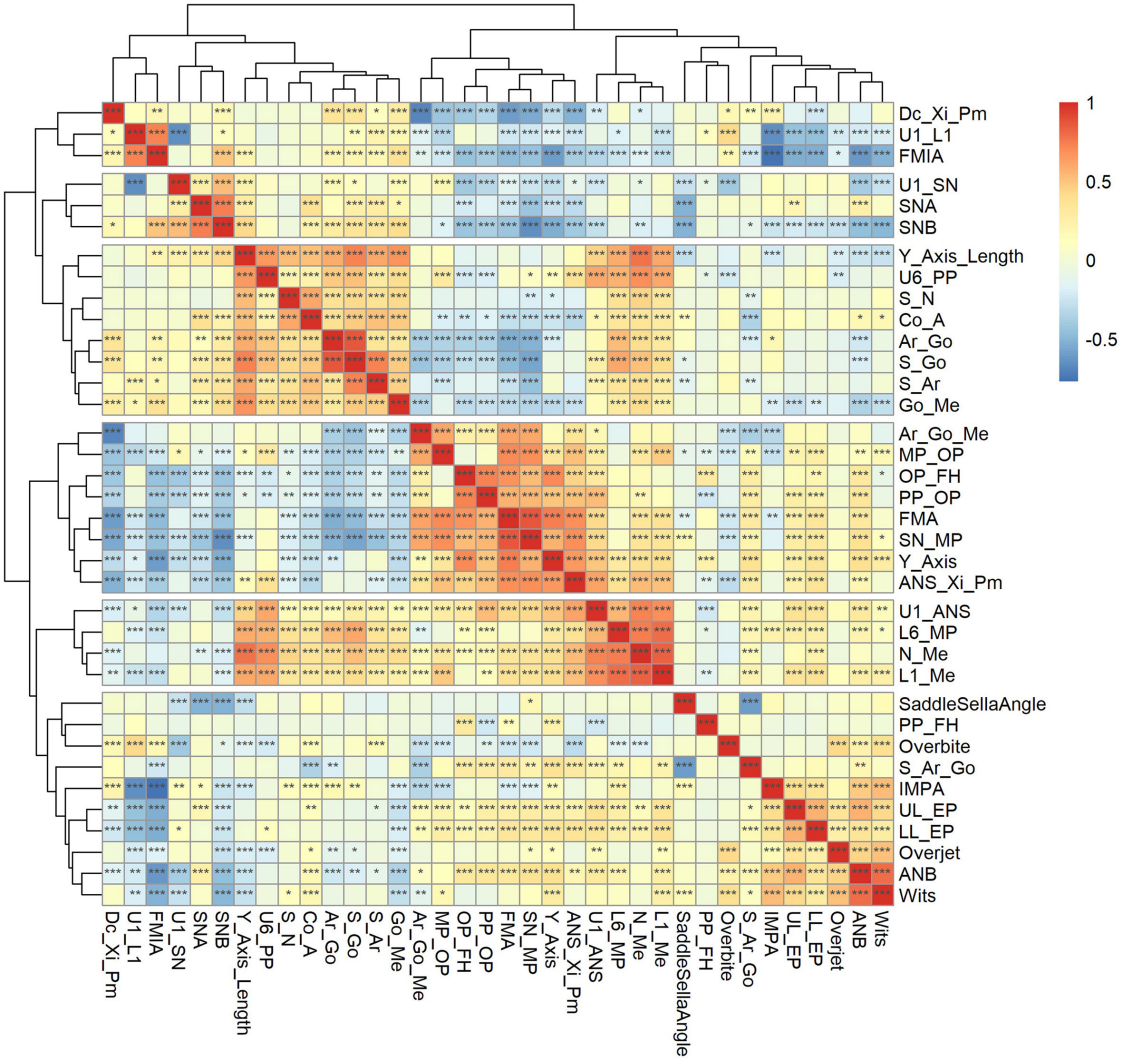
Pearson's correlation coefficient heat map and hierarchical clustering dendrogram for cephalometric variables. *P < 0.05, **P < 0.01, ***P < 0.001.

One-way ANOVA, Tukey HSD *post hoc* test, Kruskal-Wallis test, Dunn *post hoc* test and Bonferroni correction were used for hypothesis testing. Pearson's correlation coefficient was used to explore the correlation of the normalized variables in cephalometric data and was visualized by a heat map (Figure 2). The Pearson's correlation coefficient of 0.40, 0.60, and 0.80 were considered weak, moderate and strong associations respectively. At the same time, hierarchical clustering was performed on the normalized variables. Feature selection and feature transformation was conducted to improve the final clustering effect. All statistical analyses were based on Language R, version 4.1.3 (R Foundation for Statistical Computing, Vienna, Austria). *P* < 0.05 was considered statistically significant.

## Results

### Baseline characteristics of the cephalometric variables

Five hundred and one adult orthodontic patients diagnosed with TMD were included in this study. The mean age of the patients was 31.63 ± 10.56 years. Of the 501 patients, 91 were males and 410 were females (81.8%). Thirty-six cephalometric parameters shown in Table 1 were measured to reflect the TMD patients' maxillofacial features in six categories, including cranial base, maxilla, mandible, intermaxillary relation, teeth and soft tissue (Table 2).

**Table 2:** Baseline characteristics of the cephalometric variables.

**Variables**	**Male (*n =* 91)**	**Female (*n =* 410)**	**Total (*n =* 501)**
Age	29.25 (9.95)	32.16 (10.63)	31.63 (10.56)
**Cranial base**			
Saddle/Sella Angle	126.05 (5.10)	125.62 (5.29)	125.70 (5.25)
S-N	65.39 (2.91)	61.90 (2.85)	62.53 (3.16)
S-Ar	35.08 (3.50)	32.13 (2.90)	32.66 (3.22)
**Maxilla**			
SNA	82.52 (3.63)	81.87 (3.49)	81.98 (3.52)
PP-FH	0.14 (2.76)	0.19 (2.76)	0.18 (2.76)
**Mandible**			
SNB	78.28 (4.14)	77.57 (3.76)	77.70 (3.84)
Ar-Go-Me	116.91 (7.21)	117.49 (5.97)	117.39 (6.21)
Ar-Go	50.73 (5.10)	46.01 (4.15)	46.87 (4.70)
S-Ar-Go	148.69 (7.12)	151.24 (6.45)	150.78 (6.64)
Dc-Xi-Pm	37.11 (5.64)	37.01 (5.64)	37.03 (5.63)
SN-MP	31.78 (6.34)	34.53 (5.96)	34.03 (6.12)
S-Go	82.57 (6.64)	75.66 (5.38)	76.91 (6.22)
Go-Me	70.98 (6.11)	68.07 (4.39)	68.60 (4.87)
**Intermaxillary**			
Co-A	84.96 (6.63)	79.40 (4.18)	80.41 (5.18)
ANB	4.23 (2.71)	4.29 (2.69)	4.28 (2.69)
Y-Axis	60.87 (3.64)	61.29 (3.45)	61.21 (3.48)
Y-Axis length	121.40 (7.28)	114.76 (5.96)	115.96 (6.72)
Wits	1.31 (3.68)	0.53 (3.43)	0.67 (3.49)
N-Me	119.48 (6.69)	113.93 (6.13)	114.94 (6.58)
FMA	22.37 (5.83)	24.68 (5.39)	24.26 (5.54)
ANS-Xi-Pm	46.39 (4.44)	47.31 (4.73)	47.14 (4.69)
**Dental**			
IMPA	98.58 (7.70)	97.26 (7.55)	97.50 (7.59)
FMIA	59.04 (8.31)	58.04 (8.56)	58.22 (8.52)
U1-L1	126.27 (11.01)	125.72 (12.19)	125.82 (11.97)
U1-SN	103.34 (7.77)	102.46 (8.62)	102.62 (8.47)
U6-PP	23.60 (2.44)	22.38 (2.10)	22.60 (2.21)
L6-MP	33.67 (2.90)	31.48 (2.61)	31.88 (2.80)
U1-ANS	29.00 (2.94)	28.39 (2.51)	28.50 (2.60)
L1-Me	41.71 (3.11)	39.54 (3.06)	39.93 (3.18)
MP-OP	15.20 (3.86)	16.20 (4.10)	16.02 (4.08)
PP-OP	6.74 (3.69)	8.16 (3.43)	7.91 (3.52)
OP-FH	7.24 (4.14)	8.48 (3.78)	8.25 (3.87)
Overbite	2.76 (2.05)	2.52 (1.79)	2.56 (1.84)
Overjet	4.00 (1.89)	4.04 (1.66)	4.03 (1.70)
**Soft Tissue**			
UL-EP	0.81 (2.81)	0.37 (2.60)	0.45 (2.64)
LL-EP	0.98 (2.54)	0.76 (2.64)	0.80 (2.62)

Mean (SD), SD, standard deviation.

### Cluster tendency of cephalometric data

**Figure 3:**
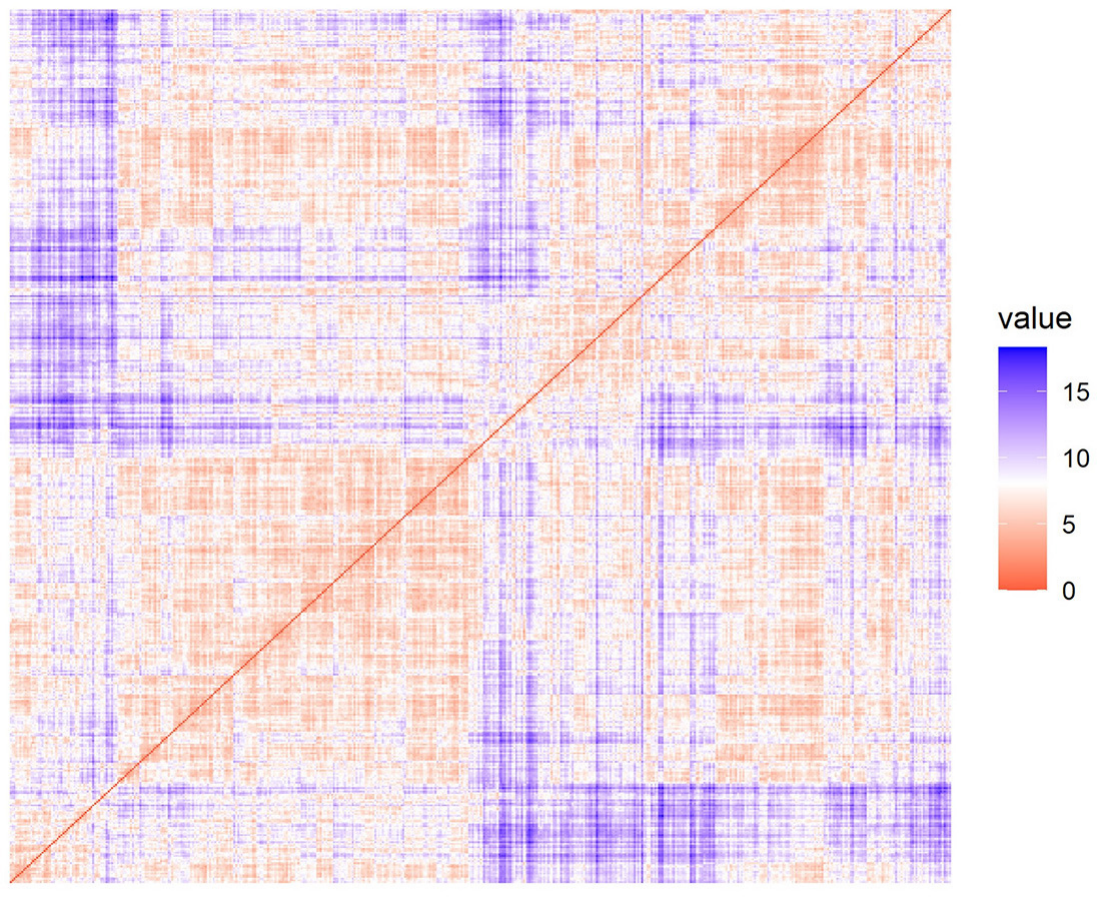
Ordered dissimilarity image for cluster tendency analysis.

According to the ODI, it was observed that the dissimilarity matrix presented a block phenomenon along the inverse diagonal direction (Figure 3), indicating that cephalometric data had a cluster tendency. The Hopkins statistic (*H* = 0.736 > 0.7) also showed a significant cluster tendency of cephalometric data, which ensured the statistical significance of clustering analysis.

### Principal component analysis for cephalometric data

A strong linear correlation was found by correlation analysis and cluster tendency analysis implied that feature selection and feature transformation should be conducted to improve the final clustering effect (Figure 2). Therefore, it was necessary to perform PCA to combine variables.

**Figure 4:**
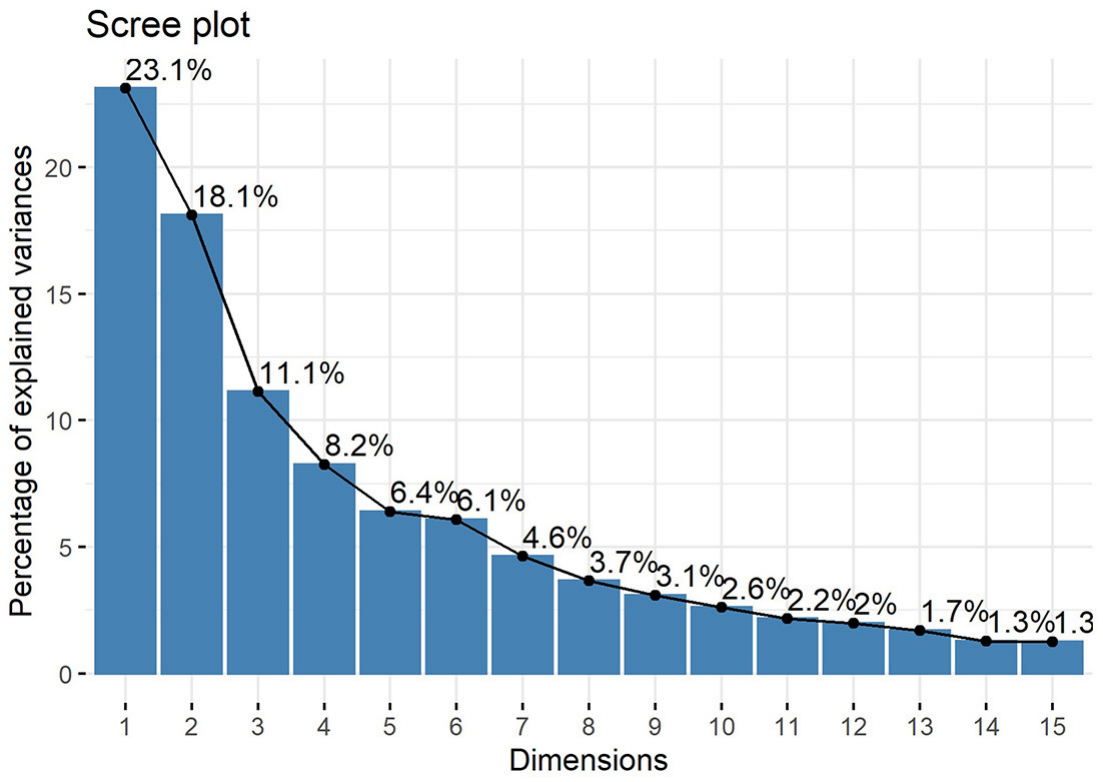
Scree plot. The values indicate the percentage of variance and show that the cumulative percentage of the variance of the first 12 PCs reached 91.2%.

**Figure 5:**
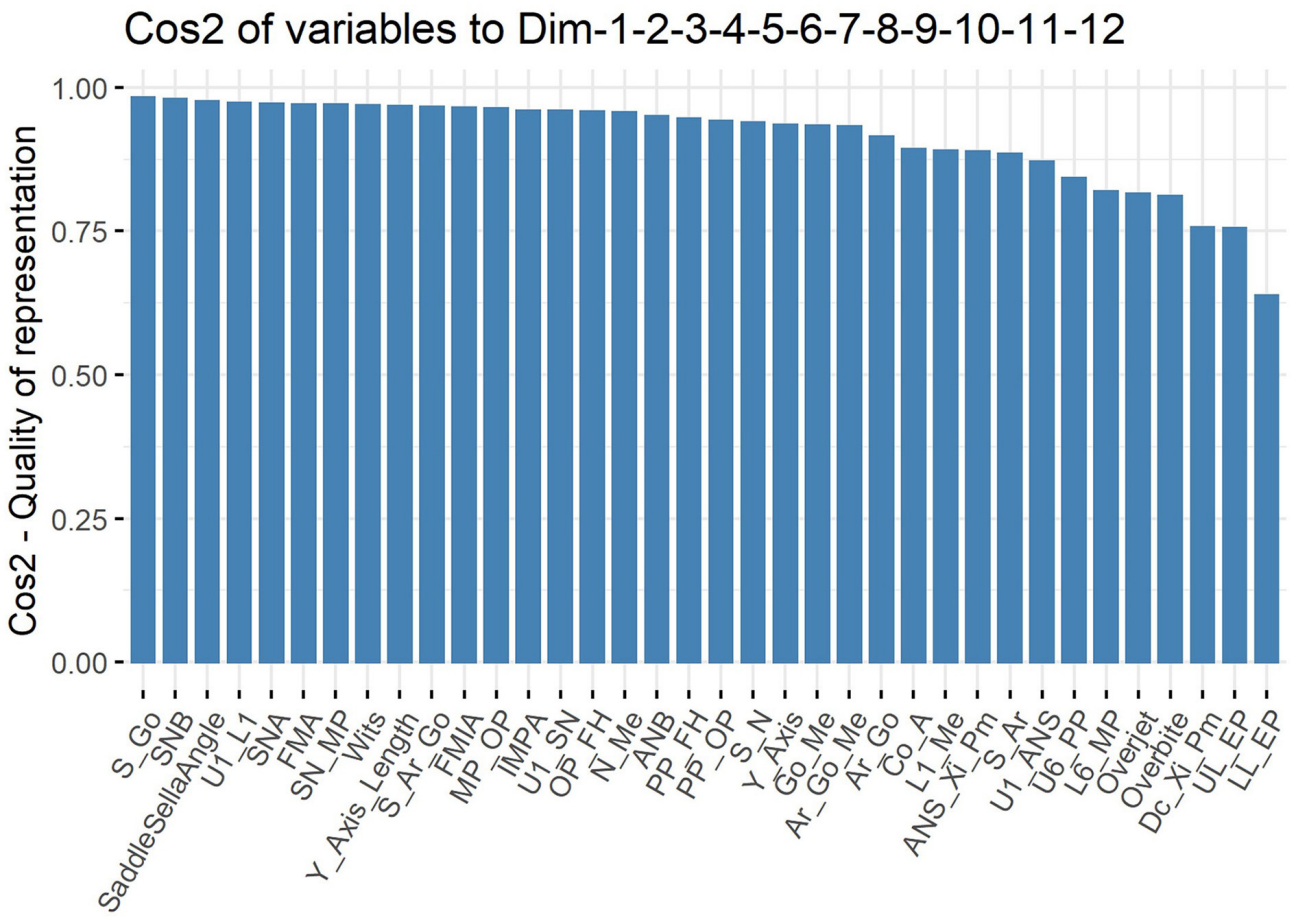
Cos2 plot. 24 of the 36 cephalometric variables had Cos2 values >0.9 (66.7%) and 33 were >0.8 (91.7%).

The cumulative percentage of the variance of the first 12 PCs was calculated to be 91.2%, and the percentage of the variance of each PC after the 13th PC < 2% (Figure 4). Consequently, the first 12 PCs were chosen to represent the entire data. The Cos2 of the first 12 PCs on each variable was calculated, and the results showed that the first 12PCs were able to represent each variable to a good extent (Figure 5).

### Clusters of cephalometric data of TMD patients

**Figure 6:**
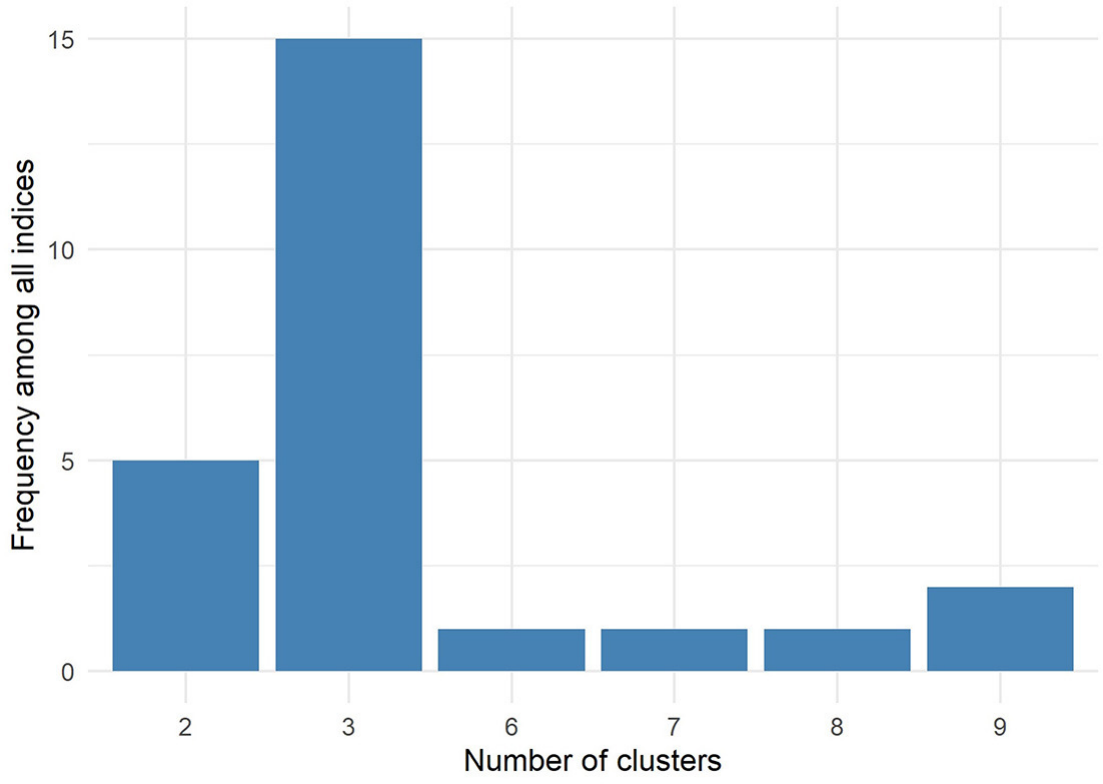
Fifteen of the twenty-six indices (57.7%) showed that the optimal number of clusters was three.

Twenty-six indices were used to evaluate different numbers of clusters for the data after principal component analysis from two to nine, and fifteen indices recommended that the data should be divided into three clusters, accounting for 57.7% (Figure 6). Connectivity, Dunn and Silhouette indices of three common clustering algorithms, including hierarchical clustering, K-means clustering and PAM, were calculated. The results (Table 3) showed that the optimal number of clusters was three and the optimal algorithm was the hierarchical clustering algorithm.

**Table 3:** Connectivity, dunn, and silhouette indices of three commonly used clustering methods in three clusters.

Indices^*^	**Hierarchical clustering**	**K-means clustering**	**PAM**
Connectivity	68.49	272.6	334.5
Dunn	0.182	0.158	0.142
Silhouette	0.152	0.152	0.100

* Dunn and Silhouette indices are positively correlated with the clustering effect while the Connectivity index is negatively correlated with the clustering effect.

**Figure 7:**
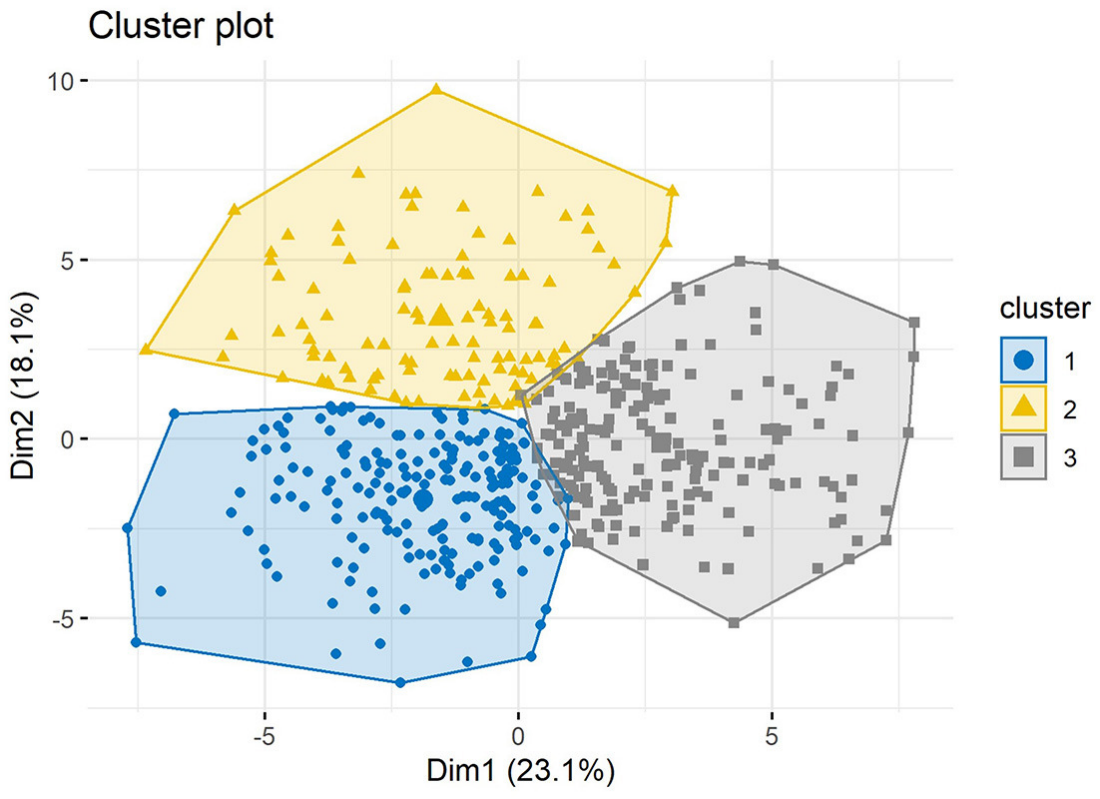
Projection of scatter plot for three clusters divided by cluster analysis on principal components. The horizontal axis represents the first principal component and the vertical axis represents the second principal component.

**Figure 8:**
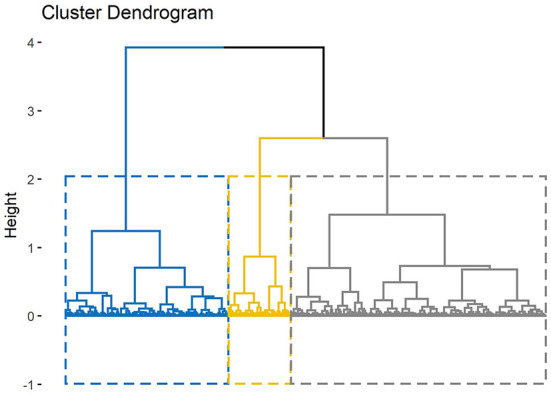
Cluster dendrogram. The height of the branches indicates the distance or dissimilarity between clusters.

Hierarchical clustering on PCs divided cephalometric data of TMD patients into three clusters which 34 of the 36 cephalometric parameters (94.4%), as well as age and sex, showed significant differences (Table 4). The projection of scatter plot for three clusters on the first two PCs (Figure 7) visualized the clustering result and the clear clustering boundaries indicated the reliability of our clustering result. The cluster dendrogram (Figure 8) visualized the clustering result from another perspective, which could show that there were no outliers in the clusters, supporting the reasonability and reliability of the clustering result.

**Table 4:** Comparison of cephalometric variables among three clusters.

**Variables**	**Cluster1** **(*n =* 202)**	**Cluster2** **(*n =* 106)**	**Cluster3** **(*n =* 193)**	***P*-value**	**Multiple** **comparisons**
Age^k^	29.83 (8.90)	31.43 (10.87)	33.63 (11.65)	0.007^**^	3 > 1
Sex(M/F)^c^	18/184	54/52	19/174	<0.001^***^	2 > (1, 3)
**Cranial Base**					
Saddle/Sella Angle^a^	125.72 (5.12)	125.20 (4.63)	125.94 (5.71)	0.511	–
S-N^k^	61.88 (2.65)	65.46 (3.23)	61.62 (2.64)	<0.001^***^	2 > (1, 3)
S-Ar^a^	32.26 (2.80)	35.83 (2.71)	31.35 (2.72)	<0.001^***^	2 > 1>3
**Maxilla**					
SNA^a^	82.37 (3.27)	83.33 (3.61)	80.84 (3.38)	<0.001^***^	2 > 1>3
PP-FH^k^	0.10 (2.62)	−0.44 (2.90)	−0.34 (2.79)	0.093	–
**Mandible**					
SNB^k^	79.09 (3.18)	79.65 (3.69)	75.17 (3.13)	<0.001^***^	(1, 2) > 3
Ar-Go-Me^k^	115.90 (5.91)	115.74 (6.87)	119.85 (5.30)	<0.001^***^	3 > (1, 2)
Ar-Go^a^	46.16 (3.64)	52.26 (4.22)	44.65 (3.53)	<0.001^***^	2 > 1>3
S-Ar-Go^k^	149.03 (6.09)	149.57 (6.19)	153.27 (6.69)	<0.001^***^	3 > (1, 2)
Dc-Xi-Pm^k^	38.60 (5.34)	38.23 (5.10)	34.73 (5.47)	<0.001^***^	(1, 2) > 3
SN-MP^k^	30.74 (4.35)	30.57 (4.70)	39.38 (4.36)	<0.001^***^	3 > (1, 2)
S-Go^k^	75.58 (4.52)	84.98 (4.77)	73.88 (4.46)	<0.001^***^	2 > 1>3
Go-Me^a^	68.42 (4.10)	73.33 (4.32)	66.18 (3.98)	<0.001^***^	2 > 1>3
**Intermaxillary**					
Co-A^k^	79.72 (4.13)	85.55 (5.42)	78.31 (4.05)	<0.001^***^	2 > 1>3
ANB^k^	3.27 (2.49)	3.67 (2.62)	5.68 (2.32)	<0.001^***^	3 > (1, 2)
Y-Axis^a^	59.07 (2.72)	60.70 (2.98)	63.74 (2.75)	<0.001^***^	3 > 2>1
Y-Axis Length^k^	113.20 (4.75)	124.65 (5.58)	114.09 (4.87)	<0.001^***^	2 > (1, 3)
Wits^k^	−0.22 (3.33)	0.52 (3.63)	1.70 (3.31)	<0.001^***^	3 > (1, 2)
N-Me^k^	109.96 (4.56)	121.42 (5.50)	116.58 (4.75)	<0.001^***^	2 > 3>1
FMA^k^	21.17 (4.14)	21.73 (4.54)	28.89 (3.97)	<0.001^***^	3 > (1, 2)
ANS-Xi-Pm^k^	43.88 (3.84)	46.92 (3.49)	50.67 (3.39)	<0.001^***^	3 > 2>1
**Dental**					
IMPA^k^	95.85 (8.24)	98.49 (7.38)	98.70 (6.66)	0.001^**^	(2, 3) > 1
FMIA^a^	62.97 (7.39)	59.77 (6.87)	52.41 (6.85)	<0.001^***^	1 > 2>3
U1-L1^a^	130.48 (11.81)	126.20 (10.99)	120.74 (10.60)	<0.001^***^	1 > 2>3
U1-SN^k^	102.91 (8.49)	104.72 (8.94)	101.17 (7.93)	<0.001^***^	2 > 3
U6-PP^a^	21.48 (1.78)	24.58 (1.78)	22.68 (2.05)	<0.001^***^	2 > 3>1
L6-MP^k^	30.02 (2.02)	34.60 (2.37)	32.34 (2.25)	<0.001^***^	2 > 3>1
U1-ANS^k^	26.57 (2.07)	29.84 (2.26)	29.78 (1.95)	<0.001^***^	(2, 3) > 1
L1-Me^k^	37.35 (2.07)	42.65 (2.54)	41.14 (2.40)	<0.001^***^	2 > 3>1
MP-OP^k^	14.13 (3.54)	15.22 (3.66)	18.44 (3.58)	<0.001^***^	3 > (1, 2)
PP-OP^a^	6.51 (3.02)	6.45 (3.03)	10.17 (3.05)	<0.001^***^	3 > (1, 2)
OP-FH^a^	7.04 (3.24)	6.56 (3.43)	10.45 (3.69)	<0.001^***^	3>(1, 2)
Overbite^k^	2.84 (1.68)	2.76 (1.93)	2.16 (1.89)	<0.001^***^	1 > 3
Overjet^k^	3.85 (1.53)	3.80 (1.79)	4.35 (1.78)	0.026^*^	–
**Soft tissue**					
UL-EP^k^	−0.82 (2.08)	0.57 (2.66)	1.73 (2.53)	<0.001^***^	3 > 2>1
LL-EP^a^	−0.58 (2.30)	0.82 (2.18)	2.24 (2.38)	<0.001^***^	3 > 2>1

Mean (SD), SD: standard deviation.

* P < 0.05,

** P < 0.01,

*** P < 0.001.

a One-way ANOVA and Tukey HSD *post hoc* test.

c Chi-square test. Bonferroni's method was used for multiple comparisons. The result showed the sex composition of Cluster 2 was significantly different from Cluster 1 and Cluster 3 with more male patients, while there were no significant differences between the sex composition of Cluster 1 and Cluster 2.

k Kruskal–Wallis test and Dunn *post hoc* test. Bonferroni's method was used for multiple comparisons.

**Figure 9:**
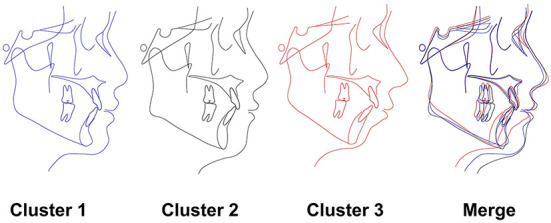
Characteristics of each cluster. The cephalometric image of the 3 subgroups as described in the results.

Patients with TMD were divided into three groups and each group could be given clinical meanings according to the cephalometrics in orthodontics as visualized in Figure 9: (a) cluster 1: skeletal class I malocclusion; (b) cluster 2: skeletal class I malocclusion with increased facial height; (c) cluster 3: skeletal class II malocclusion with clockwise rotation of the mandible and anterior open bite. Patients in cluster 1 only showed skeletal class I malocclusion (ANB = 3.27°) and normo-divergent (SN-MP = 30.74°, FMA = 21.17°). Patients in cluster 2 presented skeletal class I malocclusion (ANB = 3.67°), normo-divergent (SN-MP = 30.57°, FMA =21.73°), increased posterior facial height (S-Go = 84.98 mm), increased anterior facial height (N-Me = 121.42 mm) and a slight protrusion of upper lip (UL-EP = 0.57 mm). Patients in cluster 3 exhibited skeletal class II malocclusion (ANB = 5.68°), hyperdivergent (SN-MP = 39.38°, FMA = 28.89°), tendency of protrusive incisors (U1-L1 = 120.74°), anterior overjet (4.35 mm) and anterior open bite.

### CART model for prediction of cephalometric data category

**Figure 10:**
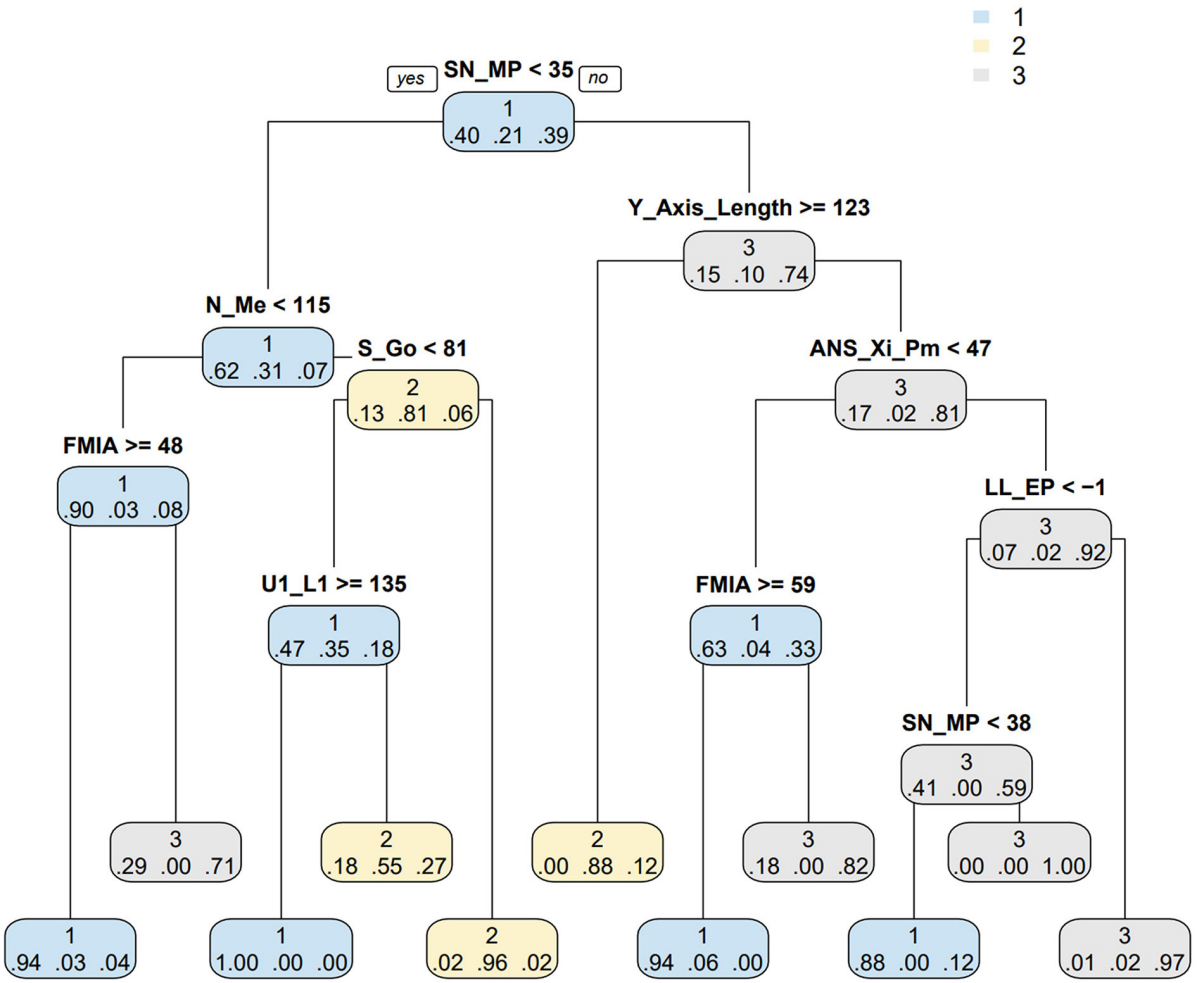
CART model for predicting cephalometric data category. The left branch of the binomial tree indicates the cases that meet the conditions while the right branch do not. The decimal below the branch indicates the probability that the sample belongs to cluster 1, 2, or 3 at this time. CART: classification and regression tree.

A CART model was built based on the clustering results (Figure 10) to easily classify TMDs into the three clusters. The data were split into training and validation set by 70: 30 and the prediction accuracy was 85.4%, which indicated the CART model had effective predictive power for our previously proposed clusters of TMD patients. Confusion matrix also showed good performance of the CART model (Supplementary Figure 1).

## Discussion

The diagnosis and classification of TMDs has been discussed since last century. However, the evaluated systems did not meet the diagnostic criteria until the Research Diagnostic Criteria for Temporomandibular Disorders (RDC/TMD) was proposed in 1992 (13). After years of expansion and refinement, the DC/TMD was released on the basis of RDC/TMD in 2014, which improved axis II procedures and delineated 12 disorders in detail. With years of practical application, the current dominant two-axis approach was not enough in clinical application and Enriqueta C. Bond noted that a broader exploration to the painful TMD beyond the two axes was necessary in future research (13). Lateral cephalometric radiograph, recognized as the most commonly used examination during orthodontal treatment (28), has been already widely applied to explore the associations between TMD and craniofacial morphology (15, 29, 30). Although the specific craniofacial features of the TMD patients were observed in many studies through cephalometric analysis, the features could not reflect the whole morphology and were difficult for clinical application. Therefore, in this study, through analyzing the features of TMD obtained from cephalometric radiograph, we developed a new category system and proposed a CART model of TMD for clinical application based on cephalometric morphology aiming to make progress for the morphological understanding of TMD. For this study was to identify the subgroups only among TMD patients, healthy populations without TMD were not included. This is the first study to classify TMD using unsupervised analysis according to lateral cephalometric radiographs in a large population (*n* = 501). The gender distribution in our study was consist with the clinical situation that females account for the majority of TMD patients (31) and the cluster analysis was conducted according to 36 morphological features, which assured a reliable and comprehensive evaluation.

In the cluster analysis, three subgroups were identified from the 36 variables among the 501 participants. In this procedure, the clustering algorithm was performed for a range of 2–9 clusters separately. According to our results, fifteen of the twenty-six indices (57.7%) showed that the optimal number of clusters was three. Intriguingly, three subgroups were also identified in another cluster analysis with a large sample including 1,031 chronic TMD cases and 3,247 TMD-free controls, which was consistent in the cluster numbers calculated in our study (32). Thus, we determined three subtypes of patients with TMD based on the cluster analysis. To our delight, each group corresponded to the entity with distinct features.

For patients in cluster 1, the values of the morphological features were mostly in the normal range (33), indicating that this group of patients did not exhibit much difference in their appearances compared with normal population, which may explain why some researchers did not find distinct relationship between morphologic features of the face and TMD when the sample size was not large enough (15). Since there was not much difference in the appearance of the patients compared with normal population in cluster 1, the TMD could be more likely developed by psychological distress than intra-articular lesion, which the latter more or less affected the morphological features of TMD patients (14, 16, 17, 34–37). Consequently, conservative therapy and psychological intervention may be the first choice for treating TMD patients in cluster 1.

Most of the cephalometric C of angles in cluster 2 were quite similar with those of cluster 1. However, the cephalometric measurements of linear distances in cluster 2 were larger than those of cluster 1, indicating cluster 2 exhibited a larger craniofacial size than cluster 1 with significant increases in posterior facial height, anterior facial height and S-Go/N-Me (70.0%). The differences may be mainly attributed to the gender factor with the percentage of males in cluster 1 and cluster 2 being 8.9 and 51% respectively. A previous study on TMD classification reported a cluster with equal gender distribution exhibited “normal” psychological conditions but were more sensitive to muscle pain (32). It can be extrapolated that patients in cluster 2 with even gender balance may also presented the same symptoms. Therefore, conservative therapy especially pain management may be optimal for treating TMD of cluster 2 for the first time. However, the validation of the abovementioned suggestion is still reserved for future work.

Specific craniofacial features observed in patients with TMD in many studies may mainly refer to the cluster 3 patients in our study (14, 16, 17). Previous studies compared the craniofacial morphology of patients with and without TMD and found that patients with TMDs exhibited specific craniofacial features such as skeletal class II malocclusion, hyperdivergent growth pattern, increased FMA, clockwise rotation of the mandible, anterior open bite and others (14, 16, 17, 34–37), reflecting the craniofacial morphology of TMD patients in cluster 3. Considering the great differences in craniofacial morphology, patients in cluster 3 may suffer from more severe TMD symptoms than cluster 1 and cluster 2. Studies revealed that the clockwise rotation of the mandible was associated with disk displacement (DD) and can be aggravated with the development of DD (38, 39). A recent study published in June 2022 suggested that the abnormality of craniofacial structures resulted from TMJ pain could be reversed by pain control therapy. Therefore, in spite of conservative therapy including pain management, it could be more important for the TMD patients in cluster 3 to improve the risky facial type. Orthodontic therapies such as passive aligners (4) or even surgical method may be considered during the treatment of TMD.

The assessment and classification of TMDs remains a challenge for dentists these days, despite multiple relevant researches in this field. This is because TMDs are a group of disease and patients can be diagnosed as multiple TMDs simultaneously due to the complex etiologies and various symptoms of TMD (7). For simple and convenient application in clinic, a CART model was designed to help dentists classify TMD according to patients' cephalometric characteristics and make a preliminary judgment of the TMD to which cluster they belonged, with the accuracy rate mostly above 80%. It will be even more easily and quickly when our category system is applied in cephalometric software with artificial intelligent analysis. In this CART model, the critical values of 8 key morphological indicators identified to distinguish among these three clusters were observed great similarity with the critical points of the cephalometrics in orthodontics. For example, the critical value of SN-MP was 35° in the CART model, which was also the critical point for distinguishing whether the mandibular plane is steep or not. The LL-EP = −1 mm in the CART model was the critical point for discriminating the retraction lower lip as well. The association reflected the accuracy and reliability of our study.

Several limitations still remained in our study. Firstly, the category system was only based on the morphological analysis, and the clinical symptoms were not involved in this system. This is because the study was a retrospective study under orthodontic background and the detailed clinical symptoms such as TMJ pain and others of the patients were not recorded. Thus, we will cooperate with the clinicians in the department of TMJ in the next step to supplement this system with clinical symptoms of TMD. Secondly, the study primarily proposed a new category system for the profile morphology of TMD, which lacked clinical verification. Further studies will be needed to verify the reliability and validity of this category system. Despite these limitations, our research creatively classified TMD according to the lateral cephalometric radiographs, which made a step toward morphological understanding of TMD.

## Conclusion

Our study primarily proposed a novel category system for the profile morphology of TMDs with 3 subgroups according to the cephalometric morphology, which dentists can easily recognize TMDs according to our CART model. This study may make a step toward the morphological understanding of TMD and benefit the management of the categorical treatment of TMD.

## Data availability statement

The raw data supporting the conclusions of this article will be made available by the authors, without undue reservation.

## Ethics statement

The studies involving human participants were reviewed and approved by the Ethics Committee of West China School of Stomatology of Sichuan University. The patients/participants provided their written informed consent to participate in this study.

## Author contributions

RZ: conceptualization, methodology, software, investigation, formal analysis, writing, and original draft. Y-HZ: data curation, writing, and original draft. Z-HZ: visualization and investigation. P-DF: resources and supervision. JW: software and validation. XX: conceptualization, funding acquisition, resources, supervision, writing—review, and editing. All authors contributed to the article and approved the submitted version.

## Funding

This work was supported by grants to XX from the Technology Innovation Project of Science and Technology Bureau of Chengdu (2022-YF05-01691-SN).

## Conflict of interest

The authors declare that the research was conducted in the absence of any commercial or financial relationships that could be construed as a potential conflict of interest.

## Publisher's note

All claims expressed in this article are solely those of the authors and do not necessarily represent those of their affiliated organizations, or those of the publisher, the editors and the reviewers. Any product that may be evaluated in this article, or claim that may be made by its manufacturer, is not guaranteed or endorsed by the publisher.

## Supplementary material

Supplementary Figure 1 Confusion matrix for CART model. Color represents the proportion of number in reference cluster. CART, classification and regression tree.
