# Supplementary figures and images for: Development of a new category system for the profile morphology of temporomandibular disorders patients based on cephalograms using cluster analysis
Source: Front Public Health. 2022 Nov 17;10:1045815. doi: 10.3389/fpubh.2022.1045815 (PMC9713943; doi:10.3389/fpubh.2022.1045815)

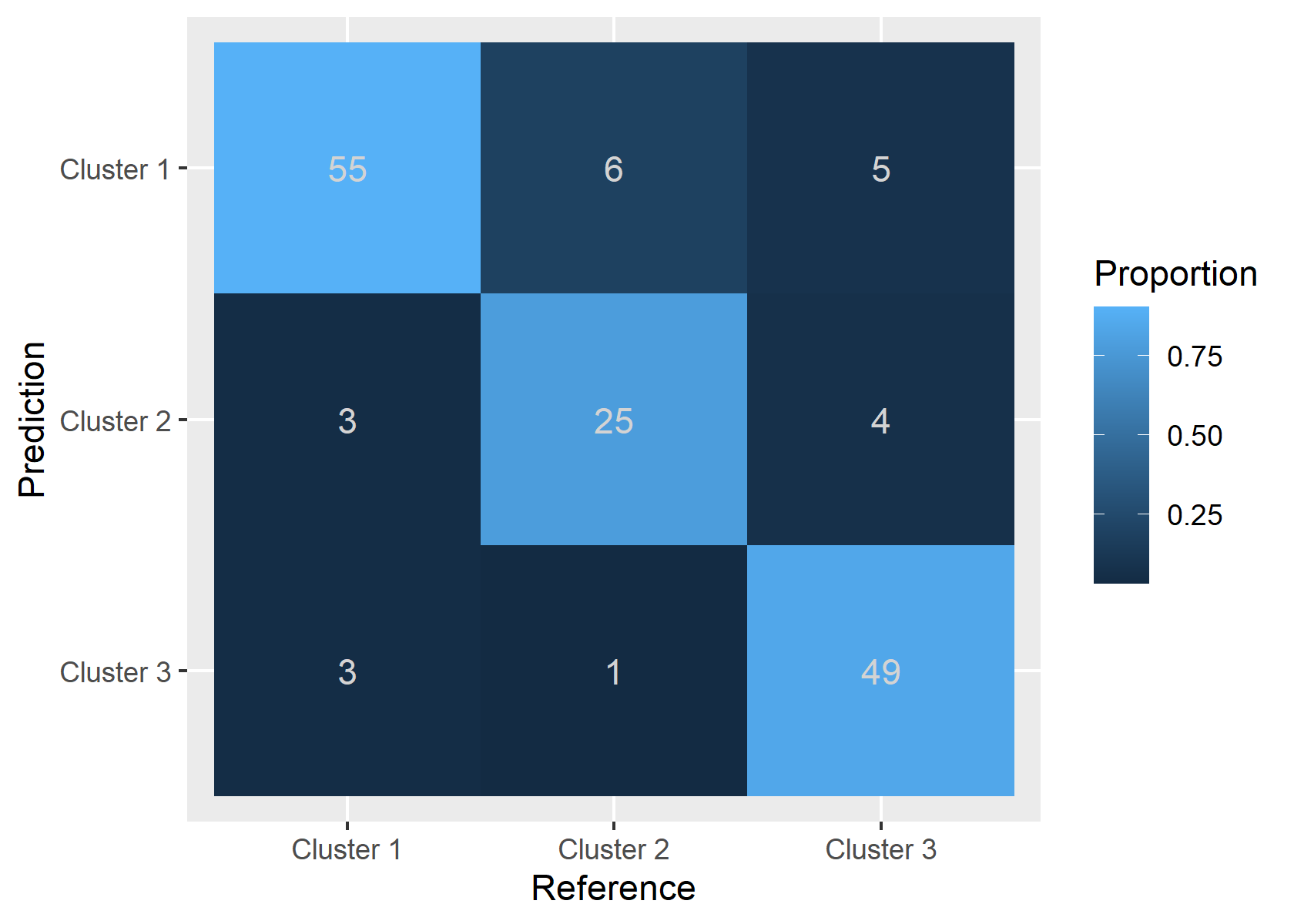

Supplement: Supplementary Figure 1 — Confusion matrix for CART model. Color represents the proportion of number in reference cluster. CART, classification and regression tree. [file Image_1.TIFF]
